# The preventive effect of sensorimotor- and vibration exercises on the onset of Oxaliplatin- or vinca-alkaloid induced peripheral neuropathies - STOP

**DOI:** 10.1186/s12885-017-3866-4

**Published:** 2018-01-10

**Authors:** Fiona Streckmann, Maryam Balke, Helmar C. Lehmann, Vanessa Rustler, Christina Koliamitra, Thomas Elter, Michael Hallek, Michael Leitzmann, Tilman Steinmetz, Petra Heinen, Freerk T. Baumann, Wilhelm Bloch

**Affiliations:** 1 Institute for Cardiovascular Research and Sports Medicine, German Sport University Cologne, Am Sportpark Müngersdorf 6, 50933 Cologne, Germany; 2 Department of Sport, Exercise and Health, University of Basel, Birsstr. 320B, 4052 Basel, Switzerland; 3 Department of Oncology, University Hospital Basel, Petersgraben 4, 4031 Basel, Switzerland; 4 Department of Neurology, University Hospital Cologne, Kerpener Straße 62, 50937 Cologne, Germany; 5 Department I of Internal Medicine, Center of Integrated Oncology Köln Bonn, University Hospital of Cologne, Kerpener Straße 62, 50937 Cologne, Germany; 6 Department of Epidemiology and Preventive Medicine, University of Regensburg, Franz-Josef-Strauss-Allee 11, 93053 Regensburg, Germany; 7 Onkologie Köln, Outpatient clinic for Hematology and Oncology, Sachsenring 69, 50677 Cologne, Germany; 8 Department of Oncology, St.Antonius-Hospital, Dechant-Decker-Str. 8, 52249 Eschweiler, Switzerland

**Keywords:** Exercise, Neuromuscular, Sensory deficits, Motor performance, Quality of life, Cancer therapy, Neurotoxic agents, Physical activity

## Abstract

**Background:**

Chemotherapy-induced peripheral neuropathy (CIPN) is a common and clinically relevant side effect of chemotherapy. Approximately 50% of all leukemia, lymphoma, colorectal- and breast cancer patients are affected.

CIPN is induced by neurotoxic chemotherapeutic agents and can manifest with sensory and/or motor deficits. It is associated with significant disability and poor recovery. Common symptoms include pain, altered sensation, reduced or absent reflexes, muscle weakness, reduced balance control and insecure gait.

These symptoms not only affect activities of daily living, subsequently reducing patients’ quality of life, they have far more become a decisive limiting factor for medical therapy, causing treatment delays, dose reductions, or even discontinuation of therapy, which can affect the outcome and compromise survival. To date, CIPN cannot be prevented and its occurrence presents a diagnostic dilemma since approved and effective treatment options are lacking.

Promising results have recently been achieved with exercise. We have revealed that sensorimotor training (SMT) or whole body vibration (WBV) can reduce the symptoms of CIPN and attenuate motor and sensory deficits. We furthermore detected a tendency that it may also have a preventive effect on the onset of CIPN.

**Methods:**

We are therefore conducting a prospective, multicentre, controlled clinical trial involving 236 oncological patients receiving either oxaliplatin (*N* = 118) or vinca-alkaloid (N = 118) who are randomized to one of two interventions (SMT or WBV) or a treatment as usual (TAU) group. Primary endpoint is the time to incidence of neurologically confirmed CIPN. Secondary endpoints are pain, maintenance of the functionality of sensory as well as motor nerve fibres as well as the level of physical activity. The baseline assessment is performed prior to the first cycle of chemotherapy. Subsequent follow-up assessments are conducted at 12 weeks, after completion of chemotherapy, and at a 3-month follow-up. Patients who develop CIPN receive an additional assessment at this time point, as it represents the primary endpoint.

**Discussion:**

We hypothesize that SMT and WBV prevent the onset or delay the progression of CIPN, decrease the likelihood of dose reductions or discontinuation of cancer treatment and improve patients’ quality of life.

**Trial registration:**

Deutsche Register Klinischer Studien (DRKS00006088, registered 07.05.2014).

## Background

Chemotherapy-induced peripheral neuropathy (CIPN) is caused by neurotoxic agents in cancer therapy. Oxaliplatin and vinca-alkaloids are two of the main agents responsible for CIPN. Oxaliplatin inhibits DNA synthesis and repair due to its ring structure, which causes the death of neural cells. Vinca-alkaloids cause axonal damage and disrupt axonal transport via microtubular damage. The main cancer patients affected by oxaliplatin are colorectal, NHL and breast cancer patients, while lymphoma patients but also ALL and pulmonary cancer patients mainly receive vinca-alkaloids. Peripheral sensory nerves are especially sensitive to toxins. Damage caused to these fibres leads to various sensory and motor dysfunctions. Patients suffer from symptoms such as loss of sensation, apparent as numbness, tingling or burning, dysaesthesia, reduced or absent Achilles tendon reflexes [1, 2] pain, and loss of balance control leading to instable gait, as well as an increased incidence of accidents and falls [3].

Even though CIPN is such a prevalent and clinically relevant side effect [4], not only diminishing patients’ quality of life, but also leading to treatment delays, dose reductions or even discontinuation of therapy, affecting the outcome and compromise survival [5], little research has been done to investigate the potentially beneficial effects of specific exercises to counteract the various motor and sensory dysfunctions.

To date, CIPN cannot be prevented and there is no consent regarding the treatment of CIPN. Research has focused on pharmacological therapies aimed at reducing CIPN or treating selected side effects while [6–8] this has been helpful for neuropathic pain, it does not address the many other side effects of CIPN [9–12]. On the contrary, many of these agents have been shown to have additional negative side effects [13]. An exercise intervention has now revealed promising results. In a first clinical trial, we [14] conducted an exercise intervention consisting of endurance, strength and sensorimotor training (SMT) twice a week for 36 weeks, accompanying lymphoma patients from diagnosis to completion of treatment. The study revealed a significant reduction of neuropathic symptoms. Patients exercising were able to reduce CIPN-related symptoms (e.g., peripheral deep sensitivity) by 87%, while in the control group no change (0%) was detected. After 36 weeks, 55% of the control group still had symptoms related to CIPN while only 4% remained with CIPN in the intervention group.

Furthermore, a positive tendency regarding the incidence of CIPN could be detected. Unfortunately, the sample size was too small in this study to show significant results. The majority of expertise on exercise and peripheral neuropathy (PNP) arises from research on patients with diabetic neuropathy. In a systematic review [15], we evaluated all exercise intervention studies for neuropathic patients independent of the cause. We found that for toxically induced PNP such as CIPN, balance exercises were most beneficial for motor as well as sensory symptoms.

Taking previous findings into consideration, this strengthened our presumption that SMT played a decisive role in the study by Streckmann et al. [14], as studies in healthy adults have revealed that SMT has the potential to counteract some of the mentioned side-effects of PNP. SMT is characterized by functional adaptations of the neuromuscular system [16, 17], regeneration of neuromuscular structures [18] and the diminished prevalence of injuries [19, 20], leading to improved proprioception [17], intermuscular coordination and balance control, causing fewer falls [21] and increasing mobility. Furthermore, studies with strength training alone or in combination with endurance training showed little to no significant intergroup differences. In line with these findings, Steimann [22] and Vogt [4] evaluated the subjective effectiveness of physiotherapy (gait training and balance exercises) and ergotherapy (e.g., walking on granulate material), while Steimann also looked at electrotherapy. Both found that patients experienced ergotherapy and physiotherapy as very helpful. One case report on a breast cancer patient, suffering from painful CIPN, showed improved balance after balance training [23].

Targeting similar mechanisms as SMT, though possibly addressing different sensory qualities, whole body vibration (WBV) has also been taken into consideration. Previous studies investigating WBV have shown a positive impact on parameters influenced by the side-effects of PNP. Kawanabe et al. [24] and Bogaerts et al. [25] showed that elderly individuals improve their gait after vibration exercises. Rittweger [26] and Kirchner et al. [27] found WBV to have a positive impact on pain reduction, while further studies showed an effect on deconditioned skeletal muscle [28], improved isometric strength [26, 29, 30], postural sway [31] and reduced fall frequency [25]. Schönsteiner et al. [32], performed a multimodal exercise program containing WBV, massage and physical exercises with CIPN patients (*N* = 131), achieving less symptoms and pain, improved physical fitness and better coordination. Both SMT and WBV require very little time and effort, but have a high impact. Especially for cancer patients, this aspect plays an important role, as therapy can be very strenuous for the patients. Training and devices are feasible, meet the requirements of hospital hygiene and are portable for all phases of therapy, even in isolation. Training is therefore even possible during cytopenias, often a limiting factor for exercise interventions concomitant to therapy.

We therefore conducted a randomized, controlled, pilot study assessing cancer patients with neurologically confirmed CIPN to either SMT (*n* = 10), WBV (*n* = 10) or a control group (n = 10) with no intervention additionally comparing them to an age- and gender matched healthy control group (n = 10). WBV and SMT were feasible for patients with CIPN and both exercise groups benefited (improved reflex activity of the Achilles- and patella tendon), peripheral deep sensitivity and pain) from 6 weeks of intervention twice a week [33].

To summarize, there are no existing prevention trials assessing the potentially beneficial effects of exercise for the onset of CIPN and only very little is known about the effects of exercise on the symptoms of CIPN. Based on our previous findings as well as from practical experience with patients, we hypothesize that SMT and WBV prevent the onset of CIPN on the one hand and/or can influence the progress of CIPN and associated motor and sensory symptoms such as balance control, coordination and mobility, as well as sensitivity, proprioception and pain, enhancing patients’ quality of life and assuring the best clinical outcome by enabling patients to receive their planned therapy regimen.

## Methods/design

### Study participants and recruitment

We plan to enrol 236 newly diagnosed haematological/oncological patients who are scheduled to receive chemotherapy containing either oxaliplatin or a vinca-alkaloid, aged ≥18 years, with the mental and physical ability to provide signed informed consent and participate in the study. Patients are recruited at three participating centres: The University Hospital of Cologne, the St. Antonius Hospital in Eschweiler and the joint practice for oncology and hematology at the Sachsenring in Cologne. Exclusion criteria is a pre-existing neuropathy of other cause. Therefore, patients will be assessed clinically for signs of neuropathy and will undergo nerve conduction studies prior to randomization. Neuropathy will be defined electrophysiologically as CMAP amplitude below 5 mV, SNAP amplitude below 5 μV, and nerve conduction velocity below 40 m/s of tibial or sural nerve). Further exclusion criteria are previous therapies containing neurotoxic agents, any contraindication for whole body vibration (instable bone metastases, acute leg thrombosis, a fracture in the lower extremities in the past 2 years, foot ulcers, artificial hips or other osteosynthesis), and myocardial infarction, angina pectoris or heart disease (NYHA III-IV) within the past six months.

### Experimental design

**Fig. 1:**
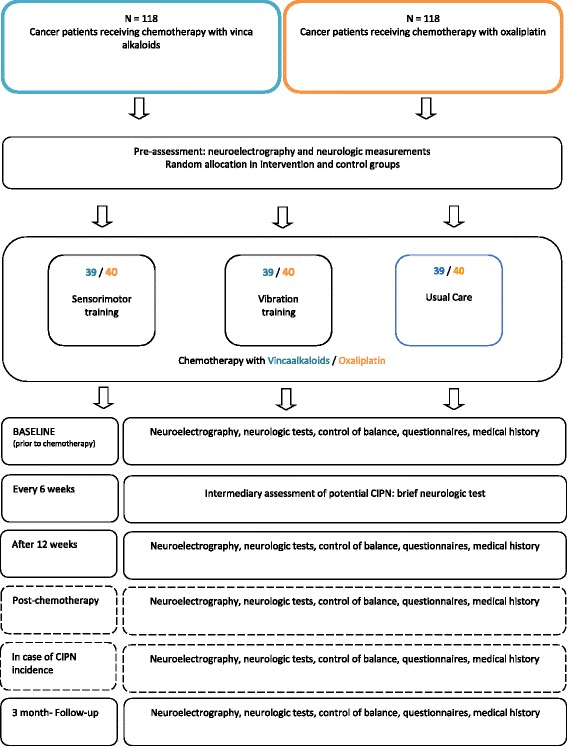
Overview of the study design

The study follows a prospective, randomized controlled design, allocating patients to three groups: an intervention group receiving SMT, an additional intervention group receiving WBV, and a control group (Fig. 1). Patients in the two intervention groups receive a defined exercise program twice a week in addition to treatment as usual (TAU). Patients in the control group receive TAU and are given the opportunity to participate in the preferred intervention after completion of the study. The interventions and assessments take place at the respective centers.

**Fig. 2:**
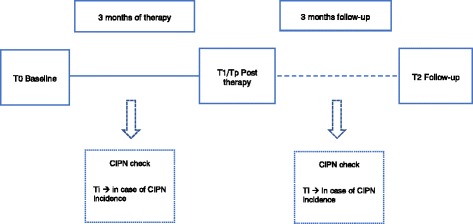
Measuring time points for patients with 3 months of therapy

**Fig. 3:**
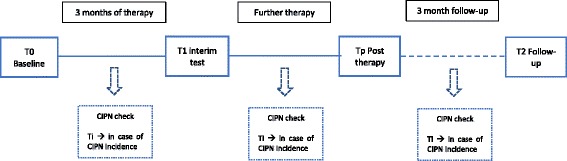
Measuring time points for patients with more than 3 months of therapy

Data is assessed at 3 to 5 measuring time points, depending on the length of medical therapy and a potential incidence of CIPN (Fig. 2 and Fig. 3). The baseline assessment (T0) is performed prior to the first cycle of chemotherapy. All patients are re-assessed after three months (T1). For most patients, this is simultaneously the post-therapy measurement (Tp) (Fig.2), while for patients who are treated for more than three months it represents an interim assessment (T1) (Fig.3), in order to ensure comparability regardless of the entity. These patients have an additional assessment upon completion of their medical therapy (~6 months). The follow-up measurement is performed three months after completion of chemotherapy (T2) in order to compensate for any potential coasting effects. Each assessment has a duration of 90 min at most. To ensure the detection of CIPN, patients are informed about possible symptoms of CIPN and asked to report back to the study coordinators immediately. Furthermore, patients are regularly asked for potential symptoms by their physicians. Additionally, a short neurological test battery is performed every 6 weeks. Sports therapists will be blinded and must not ask patients about CIPN symptoms during the interventions in order to obtain comparability with the control group. In case a CIPN is neurologically confirmed, patients are also tested at this time point (Ti).

### Assessment - primary endpoint

In order to assess the time to incidence of a neurologically confirmed CIPN, a comprehensive **Neurophysiological assessment** that includes the entire symptom pattern of CIPN, is necessary (Table 1):

**Table 1:** Flow-chart of assessments

		Baseline T0	T1	Tp	T2	Ti	Status measurement
Time points		Prior to first cycle of therapy	After 3 months	After medical therapy	3 month follow-up	Incidence CIPN	every 6 weeks
Intervention	Training	2 x per week throughout entire medical therapy			Patients offered to continue		
Anamnesis I	Entity, stadium, pre-treatment, pre-diseases, allergies, planed therapy, neurological anamnesis, CIPN relevant medication, social anamnesis	X					
Anamnesis II	Begin of CIPN Symptoms, reception of planed therapy. Amount of cycles, potential change of medication or therapy, CIPN relevant medication			X	X	X	
Anamnesis III	Reception of CIPN relevant medication, therapy of CIPN			Only CG	Only CG	Only CG	
Neurological assessment	Neuroelectrography (NCV, Amp)	X	X	X	X	X	
	Neurological clinical tests battery	X	X	X	X	X	X
Performance status	Static and dynamic postural control	X	X	X	X	X	
Questionnaires	Subjective reduction of symptoms (FACT/GOG-Ntx / EORTC CIPN 20) Quality of life (EORTC QLQ-C 30) Neuropathic pain (PainDETECT and VAS) Level of physical activity (FFKA)	X	X	X	X	X	

*CIPN* Chemotherapy-induced peripheral neuropathy, *CG* control group, *NC*V nerve conduction velocity, *Amp* amplitude, *VAS* visual analogue scale

Nerve conduction studies are performed by trained, certified and blinded examiners. For patients of the University Hospital Cologne as well as the joint practice at Sachsenring, neurophysiological assessments are performed in the Electrophysiology Laboratory of the Department of Neurology, University Hospital Cologne. Patients in Eschweiler are seen by a local neurologist. Assessment methods are standardized and aligned among the investigators. Furthermore, patients are asked not to mention the arm they are participating in to the investigators. Examiners are trained by a gold-standard examiner using a standard operating procedure and certified prior to the study.

#### Nerve conduction studies

For nerve conduction studies, motor and sensory nerves are assessed. Compound muscle action potentials (CMAP), distal motor latency, conduction velocity, and F-waves are obtained from the tibial nerve. The tibial nerve is stimulated at the ankle and popliteal fosse. Antidromic sensory nerve conduction studies are performed in the sural nerve. Sensory nerve action potentials (SNAPs) are recorded from the lateral malleolus with surface electrodes. Skin temperature is monitored and maintained above 32 °C using a heater if necessary.

We furthermore conduct a standardized neurological clinical test battery that is a feasible assessment method for oncological patients in order to check for first neuropathic symptoms. It is used as a pretest to screen for CIPN related symptoms. Should one of the components show irregularities, a neuroelectrography is required in order to detect and document a possible CIPN.

The test battery contains the following assessments:

1. **Peripheral deep sensitivity** is evaluated by the use of a Rydel-Seiffer tuning fork (128 Hz) with a scale from 0 to 8. Due to age related neural deconditioning, values ≤4 are pathological for patients ≥60 years old, while for patients under 60 years old, ≤5 is regarded as pathological [34].
2. **The Reflex action** of the Achilles- as well as the patellar tendon is assessed with a reflex hammer and graded on a 3-point scale (1 = agile, 2 = weak, 3 = missing).
3. **Sense of position** is assessed by asking patients if they can recognize a change of position in their first toe, with their eyes closed.
4. **Perception of touch** is evaluated by symmetrically stroking the outsides of the patients’ legs and feet in order to detect reduced or altered sensation due to demyelination or axonal degeneration [30, 35].
5. **The strength of the lower leg muscles** is assessed by requesting the patient to actively move their legs against the resistance of the examiner’s arm. The examiner then grades the strength on a six-point scale (0 = no activity, 1 = visual contraction without motor effect, 2 = movement under elimination of gravity, 3 = movement under gravity, 4 = movement against slight resistance 5 = normal force).

### Assessment – Secondary endpoints

#### Postural control

A force plate (Leonardo Mechanograph®, Novotec medical, Pforzheim, Germany) is used to assess changes in the center of pressure during upright static and dynamic stance. The assessment follows a standardized protocol (see Table 1). Primarily, the supporting foot is determined with a short test [36]. Patients are asked to maintain an upright position with their knees slightly flexed (~30°), hands at their side and their gaze straight ahead for 30 s. The cumulative change in sway paths during this period is registered and serves as a measure of postural control. To minimize bias through potential learning effects, each position is repeated three times. Additionally, failed attempts are recorded should a patient seek hold. The tasks become progressively more difficult as previous studies (see reference [37] for review) have shown that postural tasks with different complexity serve best to test for changes in stance stability after balance training. To assess the dynamic stance, a balance pad is additionally placed on top of the force plate.

#### Questionnaires

##### FACT/GOG-Ntx - questionnaire

The particular sector of the FACT/GOG-Ntx [Functional Assessment of Cancer Therapy/Gynaecology Oncology Group – Neurotoxity] is used to document and assess the severity of the subjective PNP symptoms [38]. This questionnaire has been validated and contains eleven items which allow an assessment of the extent of PNP symptoms – from “not at all” to “very much” [25].

##### EORTC-QLQ-CIPN20

The EORTC-QLQ-CIPN20 is a phase IV questionnaire that we are evaluating for N. Aaronson in the course of this study. It is a 20-item questionnaire that was developed to elicit patients’ experience of symptoms and functional limitations related to CIPN.The CIPN20 has 3 subscales: a sensory, a motor, and an autonomic subscale.

##### EORCT-QLQ-C30

The EORTC-QLQ-C30-questionnaire is used to assess health-related quality of life. In addition to a scale for “global quality of life”, the questionnaire contains five functional scales (physical, emotional, social and cognitive functions, and role functions), three symptoms scales (fatigue, pain, nausea/vomiting), and single item scales of respiratory distress, insomnia, loss of appetite, constipation, diarrhoea, and financial problems. The questionnaire has been validated and translated into 81 languages and has been used in more than 3000 studies worldwide. It is internationally regarded as reliable [39, 40].

##### PainDETECT

This questionnaire focuses on pain specifically related to PNP. It helps assess patients’ subjective experience of neuropathy-related pain. The questionnaire includes 12 items that take the intensity, progression, and distribution of pain into account. The questions are answered on a Likert scale ranging from “not at all” to “very much”, which are summed up to yield a total score that reflects neuropathic pain status. Pain DETECT is a validated and reliable screening tool with high sensitivity, specificity and positive predictive accuracy [41, 42].

##### FFKA

Physical activity levels are evaluated using the Freiburger Physical Activity Questionnaire (FFKA), a standardized and validated questionnaire that assesses the physical activities performed by a patient during the past 4 weeks. Based on the patients’ answers, MET-scores are calculated [43, 44].

See Table 1 for Flow-chart of all assessments.

### Training program

The interventions start immediately after randomization and are continued throughout the entire medical therapy (~3 to 6 months). Training sessions are supervised and take place twice a week in specific training rooms designed to meet the needs of oncological patients in an outpatient setting or during the hospital stay, in one of the centers. Each session lasts for about 15 to 30 min. Depending on the type of intervention, the training will involve:

- **Sensorimotor training** consists of progressively more difficult balance exercises on progressively instable surfaces. Each patient performs 4 exercises per session following a standardized protocol (see Table 1). Each exercise is performed three times for 20 s, allowing a 40 s rest between each set and a 3-min rest between each exercise, to avoid neuronal fatigue. Patients are asked to stand barefoot or in socks, their foot in a previously acquired “short-foot-position”, knees slightly flexed (30°), and to maintain balance.
- **Vibration training** takes place on a vibration platform (Galileo™, Pforzheim, Germany)®. Each training session consists of four sets of 30 s to 1 min vibration. The frequency of the vibrating platform ranges between 18 and 35 Hz with a 2 mm amplitude. Between sets, the patients rest for at least 1 min to avoid fatigue. Patients are asked to stand on the platform barefoot and on their forefeet or if they are too instable, an 80/20% distribution of weight on the forefeet rather than the heels.

Each training session allows for individual progression within a standardised selection of exercises (see Table 1) and is documented by the sports therapist.

### Statistical procedures and sample size estimation

Central computerized randomisation (RITA) using a modified minimization procedure with stochastic component according to Pocock and Simon is performed [45]: intervention 1: intervention 2:control = 1:1:1, stratified by study center and type of therapy (Oxaliplatin, Vinca-alkaloids). In trials under similar conditions, a balanced randomization has been achieved using this algorithm [14].

Sample size calculation is based on the primary endpoint incident CIPN. Power calculation is based on the following scenario: The assumed incidence rate with TAU is 90%, which was informed by a review of the literature. In both intervention groups, we assumed an incidence rate of 75%. The effect size corresponds to a relative risk of about 0.60, which is a clinically meaningful effect size. Using the log-rank test (1-β = 0.8, two-sided α = 0.05), we need a total of 196 evaluable patients, 65 per group [46, 47]. We anticipate a drop-out rate of 10%, yielding a total of 236 patients to be recruited for this study, 79 per group. This calculation is conservative as we may achieve additional power performing the final analysis using a multivariable Cox proportional hazards regression model adjusting for study center, type of chemotherapy, type of cancer, gender, and age.

#### Recruitment of patients

Patients are recruited in three centers: The University Hospital Cologne/ CIO Cologne Bonn, the St.-Antonius-Hospital in Eschweiler, and the Oncological Practice at the Sachsenring in Cologne. The numbers of patients are based on the average number of patients in the respective centers per year over the past 2 years, considering denial or drop-out and applied to the recruitment period.

#### Data management and analyses

Data entry is continuously monitored by a data manager (TN) and will be analyzed by a statistician (ML). For the primary endpoint incident CIPN, censoring will be taken into account by using log-rank tests, and multivariable Cox proportional hazard regression models will be used to test for differences between groups and to estimate treatment effects. For categorical secondary endpoints, Fisher’s exact test and Wilcoxon signed-rank tests and multivariable logistic regression models will be performed. For continuous outcomes (including scores derived from self-report questionnaires), t-tests and multivariable linear or median regression models will be used. Multivariable models will adjust for study center, type of chemotherapy, type of cancer, gender, and age. Intention-to-treat analyses will be conducted based on complete cases and on multiply imputed data using a conditional imputation [48].

## Discussion

### Expected key results

To date there is no prevention or effective treatment for neuropathies though it presents a diagnostic dilemma as physicians need to find the balance between patients’ quality of life and the effectiveness of medical therapy. Our main study aim is therefore to evaluate the potential of sensorimotor training and whole-body vibration to prevent CIPN. We expect that both interventions (SMT and WBV) will be able to prevent or at least postpone the incidence of CIPN and in case of occurrence at least reduce the severity of subjective and objective CIPN-related symptoms such as loss of peripheral deep sensitivity, pain, weakened or absent reflexes or loss of balance control, enabling patients to receive their planned medical therapy. A successful implementation would therefore be of high clinical relevance.

### Benefits and risks

Patients have the potential benefit of being able to prevent the incidence of CIPN or at least reduce their debilitating symptoms of CIPN without any further side-effects. We do not expect any complications. The interventions have no negative influence on their medical therapy. All groups receive the best medical standard. However, we have to account for the possibility that patients with neuropathic pain in the lower extremities may possibly experience some pain during the vibration exercises at higher frequencies. Due to the low submaximal intensity, the position taken on the platform, and the well-established, non-invasive assessment methods, we believe the possible risk is very low for patients. The electroneurography is a neurological routine assessment that is not associated with any specific risk. Due to the fact that electricity is used, it is possible that some patients may experience this sensation as uncomfortable or painful.

### Potential for bias

In an exercise intervention study, where patients have to be trained and supervised by qualified exercise therapists, patients are aware of their allocation to the treatment or control group. It is therefore essential that investigators performing the assessments are blinded as to which arm patients are in and are not allowed to train the patients and vice versa. All measurements are performed using highly standardized procedures. Assessments are standardized as well as aligned among the investigators. Patients will additionally be asked not to reveal the result of randomisation to any investigator except of course to the exercise therapist. The study can therefore be considered single-blinded. To further reduce bias, all three centres are equipped with identical technology enabling optimal conditions for comparable data collection. The study coordinator (FS) is the same for all study centres and training of study assistants is identical. All assessments within an individual are always performed by the same trained investigator. Assessments are performed according to standardized operating procedures, at the same time of day, in the same room and maintaining a consistent temperature. Regular meetings are held to optimize coordination of data collection and collaboration among the study centres. Follow-up measurements will be carried out by investigators who are unaware of the treatment allocation, resulting in an unbiased assessment of the outcome. A randomized study design will essentially rule out confounding.

#### Perspectives

Our results may contribute to improved supportive care in oncology, thereby enhancing quality of life, enabling the optimal medical therapy in neuropathic cancer patients and, eventually, possibly even improving survival for these patients.

We furthermore expect that the proposed interventions will lead to an improvement of motor and sensory functions (such as balance control, coordination, sensitivity, reflexes, pain) impacted by CIPN. It will help understand the underlying mechanisms of SMT and WBV on motor and sensory functions impaired by PNP. It could assure best clinical outcomes by improving the side-effects of CIPN without interfering with the planned therapy regime, impacting supportive care for cancer patients. Patients’ mobility, autonomy and activities of daily living could be maintained. Consequently, patients’ quality of life would be increased. Further possible side-effects (e.g., fatigue) could be decreased and secondary diseases reduced. Additionally, patients’ social reintegration could be enhanced. The results can help develop recommendations for patients suffering from CIPN, improving supportive care for cancer patients.

We furthermore aim at publishing the results in peer-reviewed scientific journals, raising the awareness of the scientific community for this topic. Furthermore, we will create guidelines, training recommendations, and manuals for clinical practice and health care professionals that can directly be translated into patients’ everyday lives. Finally, our results will form the foundation for future research on this topic.

## Abbreviations

CIPN: Chemotherapy-induced peripheral neuropathy
EORTC-QLQ-C30: European Organisation for Research and Treatment of Cancer – Quality of Life Questionnaire – 30 item core questionnaire
EORTC-QLQ-CIPN20: European Organisation for Research and Treatment of Cancer – Quality of Life Questionnaire – 20 item CIPN-specific questionnaire
FACT-GOG-Ntx: Functional Assessment of Cancer Therapy/Gynaecology Oncology Group – Neurotoxity
FFKA: Freiburger Fragebogen für Körperliche Aktivität – level of physical activity questionnaire
PNP: peripheral neuropahy
SMT: Sensorimotor training
WBV: whole-body vibration
